# Hemostasis After Brachial Artery Access With the MynxGrip Device: A Case Report

**DOI:** 10.1177/1179546818759298

**Published:** 2018-02-20

**Authors:** Arturo Giordano, Stefano Messina, Gennaro Maresca, Giuseppe Biondi-Zoccai

**Affiliations:** 1 Unità Operativa di Interventistica Cardiovascolare, Presidio Ospedaliero Pineta Grande, Castel Volturno, Naples, Italy; 2 Unità Operativa di Emodinamica, Casa di Salute Santa Lucia, S. Giuseppe Vesuviano, Italy; 3 Department of Medico-Surgical Sciences and Biotechnologies, Sapienza University of Rome, Latina, Italy; 4 Department of AngioCardioNeurology, IRCCS Neuromed, Pozzilli, Italy

**Keywords:** Arteriotomy closure device, brachial artery, case report

## Abstract

**Purpose:**

Brachial access is occasionally used for endovascular procedures when other more established or safer ones (eg, femoral or radial) are contraindicated. Although manual compression is the standard of care after brachial arteriotomy, suboptimal compression may lead to bleeding or thrombosis. Arteriotomy closure devices have thus been proposed as an alternative means to achieve hemostasis after brachial arteriotomy. Yet, there is a paucity of evidence and a limited spectrum of devices suitable for brachial arteriotomy closure. We present the use of the MynxGrip closure device after brachial arteriotomy.

**Case:**

A 70-year-old gentleman underwent brachial arteriotomy followed by iliac artery stenting with a 7F compatible device. Hemostasis was then achieved with the MynxGrip device, uneventfully.

**Conclusions:**

This clinical vignette highlights the feasibility and safety of brachial use of the MynxGrip device in experienced hands, suggesting that it can represent a useful adjunct to the armamentarium of the endovascular specialist.

## Introduction

The brachial artery has been historically a common access site for endovascular procedures.^1^ Miniaturization of arteriotomy kits and revascularization devices has lead to a shift toward femoral and radial accesses,^2^ but brachial access is still occasionally used when femoral or radial access is absolutely or relatively contraindicated.^3^ Despite its superficial track, brachial puncture is more challenging than femoral puncture, given the proximity of veins and nerves, the common tortuosity, and the limited underlying bone surface to perform adequate manual compression. Moreover, hemostasis requires utmost care, as suboptimal manual compression may lead to severe thrombotic or bleeding complications, and even compartment syndrome, especially in anticoagulated patients.^4^ Accordingly, vascular closure devices may prove useful if carefully used in this vascular niche. Yet, given the superficial nature of the brachial artery, plug-based hemostasis devices may be contraindicated given their increased risk of embolization and abrupt thrombosis. In an effort to expand the armamentarium of endovascular specialists aiming at brachial hemostasis, we hereby report on a case of brachial arteriotomy closure with the MynxGrip device (Cordis; Miami Lakes, FL, USA).^5^

## Case

A 70-year-old gentleman was admitted to our institution for invasive angiography given Rutherford class 5 peripheral artery disease. He reported a history of arterial hypertension, dyslipidemia, smoking, diabetes mellitus, obesity, prior myocardial infarction, and coronary artery bypass grafting. His medications included aspirin, clopidogrel, bisoprolol, atorvastatin, oral antidiabetic agents, and insulin. Noninvasive duplex ultrasound had showed total occlusion of the right common iliac artery, significant stenosis of the left common iliac artery, and total occlusion of the left superficial femoral artery.

**Figure 1.**
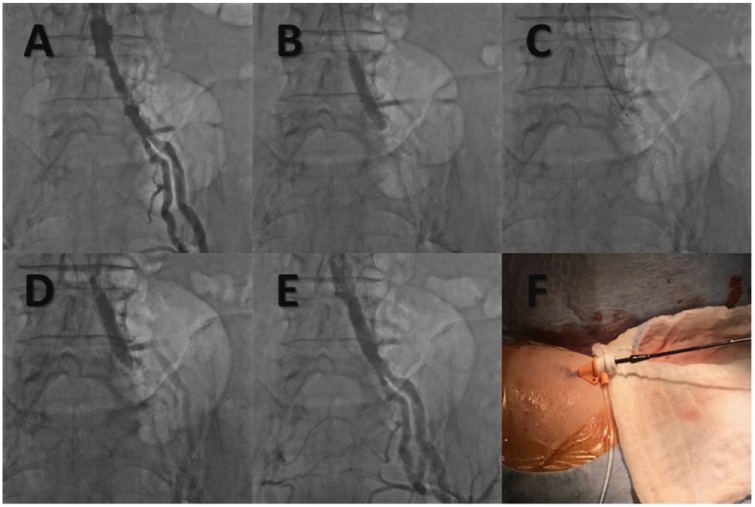
Left common iliac artery revascularization through the right brachial artery, with hemostasis achieved with the MynxGrip arteriotomy closure device. (A) Baseline angiography showing right common iliac artery occlusion and left common iliac artery stenosis. (B) Predilation with a 7.0 mm × 40 mm balloon. (C) Deployment of a 8.0 mm × 40 mm self-expanding stent. (D) Postdilation with a 7.0 mm × 40 mm balloon. (E) Final angiographic result. (F) Deployment of the MynxGrip device in the right brachial artery.

The right brachial artery was accessed with a short 6F sheath (Avanti+; Cordis), and diagnostic angiography was performed with a JR4 diagnostic catheter (Infiniti; Cordis), confirming right common iliac artery occlusion, and significant stenosis of the left common iliac artery (Figure 1). After upsizing to a 7F sheath (Avanti+), a 7F JR4 guiding catheter was placed in the distal abdominal aorta (Vista Brite Tip; Cordis), and the lesion in the left common iliac artery was crossed with a 0.014″ Pilot 150 guidewire (Abbott Vascular, Santa Clara, CA, USA). The lesion was then predilated with a 7.0 mm × 40 mm semicompliant balloon (Aviator Plus; Cordis), a 8.0 mm × 40 mm self-expandable nitinol stent (Precise; Cordis) was deployed, finally postdilated with a 7.0 mm × 40 mm semicompliant balloon (Aviator Plus), achieving a satisfactory final angiographic result.

Hemostasis in the brachial artery was then achieved with the MynxGrip device, deployed as per instructions of use. Specifically, the device was inserted into the sheath (ensuring that the uninflated balloon amply overcame the proximal tip), the balloon was inflated and gently pulled until resistance was felt (ensuring that the balloon was seated on the tip of the sheath), the whole system was retrieved until additional resistance was felt (ensuring that the balloon was not seated at the arteriotomy site), the procoagulant matrix (polyethylene glycol) was pushed in contact with the external surface of the artery, the sheath was removed during continuous balloon inflation, the procoagulant matrix was compressed by pushing the MynxGrip shaft, and the sealant was aptly deployed. The balloon was finally deflated and removed, and manual hemostasis continued for 1 minute. Radial pulse persisted throughout and up to patient discharge on the following day, without any evidence of bleeding or other access site complications. No duplex ultrasound control was performed as per our standard practice. However, the brachial, radial, and ulnar pulses were all valid at discharge.

## Discussion

We hereby report on a unique case of brachial arteriotomy managed with the MynxGrip device. Accordingly, this clinical vignette highlights the feasibility of this access closure device after brachial artery access.

Management of vascular access entails complex decision making, spanning from access site choice to sheath size selection, and eventually means to obtain hemostasis.^6^ Although brachial access is less commonly chosen in current endovascular practice, it still may be a valid option when femoral or radial access is absolutely or relatively contraindicated, such as patients with severe ilio-femoral disease requiring insertion of large sheaths.^4^ Despite its historical prominence and the ease of puncture, the brachial artery requires careful management after the procedure has been completed, as suboptimal manual compression (either too weak or too forceful) may lead to disastrous complications, ranging from severe bleeding leading to compartment syndrome to thrombosis.

Arteriotomy closure devices represent an interesting alternative to manual compression after brachial artery access.^7^ Although a good front wall puncture is a key prerequisite and a meticulous technique is paramount, favorable results have already been reported for several devices, including the AngioSeal,^8^ Boomerang,^9,10^ Perclose,^11^ and StarClose,^10,12^ among others.

Despite nonrandomized studies questioning the safety of prior generation Mynx arteriotomy closure devices,^13^ the new-generation MynxGrip device appears to have remarkably improved its ease of use and safety profile while maintaining the key pros of its predecessors.^14^ In particular, MynxGrip is based on a combination of temporary balloon occlusion of the arteriotomy and simultaneous extravascular deployment of a procoagulant matrix. Accordingly, its extravascular collagen patch with a foot pad–type grip technology may contain breakthrough bleeding; it is well contained and decreases the chance for compartment syndrome; its likelihood of distorting vessel anatomy is minimal (at odds with suture-based devices) and does not leave behind any rigid foreign material (at odds, for instance, with AngioSeal). Our case is in agreement with other anecdotal evidence on the use of the MynxGrip to achieve hemostasis after a low (mid forearm) left brachial artery access in a patient with critical limb ischemia).^15^ Accordingly, this device can be envisioned for brachial arteriotomy closure, provided an accurate front wall puncture is made and meticulous technique is employed when using the MynxGrip. Indeed, duplex ultrasound guidance and control can be a useful adjunct to maximize the safety of this device, especially when experience with it is more limited.

In conclusion, notwithstanding the clear limitations of a single case report, we consider the MynxGrip device as a useful adjunct to the endovascular specialist’s armamentarium for arteriotomy closure.
